# Common genetic variation in the glucokinase gene (*GCK*) is associated with type 2 diabetes and rates of carbohydrate oxidation and energy expenditure

**DOI:** 10.1007/s00125-014-3234-8

**Published:** 2014-04-13

**Authors:** Yunhua L. Muller, Paolo Piaggi, Duncan Hoffman, Ke Huang, Brittany Gene, Sayuko Kobes, Marie S. Thearle, William C. Knowler, Robert L. Hanson, Leslie J. Baier, Clifton Bogardus

**Affiliations:** Phoenix Epidemiology and Clinical Research Branch, National Institute of Diabetes and Digestive and Kidney Disease, National Institutes of Health, 445 North 5th street, Phoenix, AZ 85004 USA

**Keywords:** Carbohydrate oxidation, Energy expenditure, *GCK*, Thermic effect of food, Type 2 diabetes

## Abstract

**Aims/hypothesis:**

Glucokinase (GCK) plays a role in glucose metabolism and glucose-stimulated insulin secretion. Rare mutations in *GCK* cause MODY. We investigated whether common variation (minor allele frequency ≥0.01) in *GCK* is associated with metabolic traits and type 2 diabetes.

**Methods:**

Four exonic single-nucleotide polymorphisms (SNPs) and three SNPs predicted to cause loss of promoter function were identified in whole-genome sequence data from 234 Pima Indians. These seven tag SNPs and rs4607517, a type 2 diabetes variant established in other studies, were analysed in 415 full-heritage non-diabetic Pima Indians characterised for metabolic traits, and 7,667 American Indians who had data on type 2 diabetes and BMI.

**Results:**

A novel 3′ untranslated region (3′UTR) SNP, chr7:44184184-G/A, was associated with the rate of carbohydrate oxidation post-absorptively (*β* = 0.22 mg [kg estimated metabolic body size (EMBS)]^−1^ min^−1^, *p* = 0.005) and during a hyperinsulinaemic–euglycaemic clamp (*β* = 0.24 mg [kg EMBS]^−1^ min^−1^, *p* = 0.0002), the rate of carbohydrate oxidation in a respiratory chamber (*β* = 311 kJ/day, *p* = 0.03) and 24 h energy expenditure, which was attributable to the thermic effect of food (*β* = 520 kJ/day, *p* = 3.39 × 10^−6^). This 3′UTR SNP was also associated with diabetes (OR 1.36, 95% CI 1.11, 1.65, *p* = 0.002), where the A allele (allele frequency 0.05) was associated with a lower rate of carbohydrate oxidation, lower 24 h energy expenditure and higher risk for diabetes. In a Cox proportional hazards model, a rate of insulin-stimulated carbohydrate oxidation lower than the mean rate at baseline predicted a higher risk for developing diabetes than for those above the mean (hazard rate ratio 2.2, 95% CI 1.3, 3.6, *p* = 0.002).

**Conclusions/interpretation:**

Common variation in *GCK* influences the rate of carbohydrate oxidation, 24 h energy expenditure and diabetes risk in Pima Indians.

**Electronic supplementary material:**

The online version of this article (doi:10.1007/s00125-014-3234-8) contains peer-reviewed but unedited supplementary material, which is available to authorised users.

## Introduction

Glucokinase (GCK) is a hexokinase isozyme (hexokinase IV) that catalyses glucose to glucose-6-phosphate (G6P) and is involved in the first step of both glycolysis and glycogen synthesis. GCK is predominantly expressed in hepatocytes and pancreatic beta cells, with isoforms distinct in the N terminus. The pancreatic beta cell isoform is a key enzyme in regulating glucose-stimulated insulin secretion and is considered to be a glucose sensor. The liver isoform plays a central role in regulating glucose homeostasis and is a major component of the hepatic glucose-sensing system involved in glucose synthesis, breakdown and storage [1–3]. Rare heterozygous inactivating mutations in *GCK* cause MODY, mainly due to a reduced glucose-stimulated insulin secretion [4]. While rare mutations in *GCK* cause MODY, common variants have been associated with HbA_1c_ levels, fasting glucose concentrations and type 2 diabetes in white and other populations [5–7]. No rare coding variants in *GCK* were identified in 234 Pima Indians with whole-genome sequence data (unpublished data, Y. L. Muller). Thus, in the current study, we investigated the effects of common and low-frequency *GCK* variants with a minor allele frequency (mAF) ≥0.01 on metabolic traits and type 2 diabetes risk in Pima Indians.

## Methods

### Participants with outpatient longitudinal data on type 2 diabetes and BMI

**Fig. 1:**
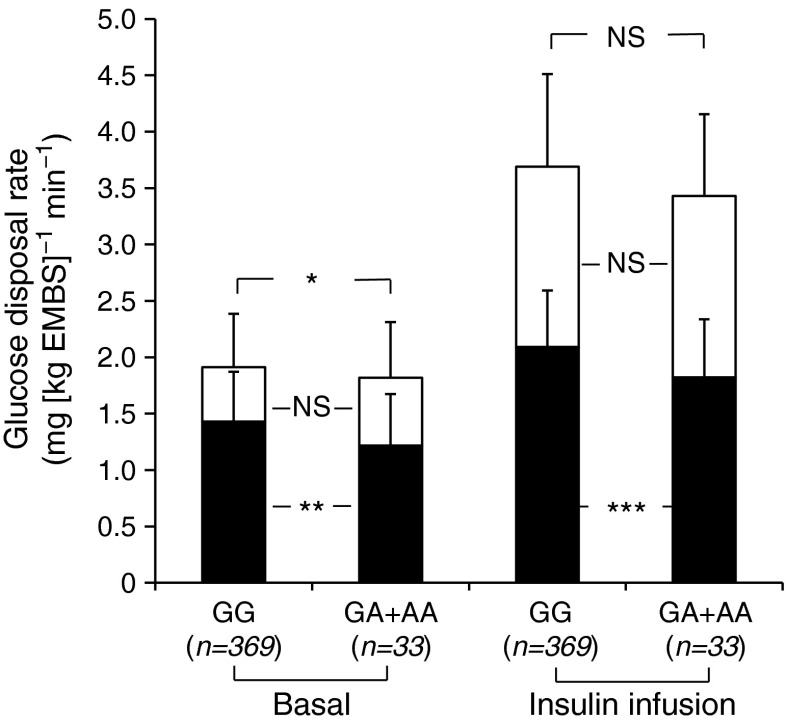
Oxidative and non-oxidative glucose disposal rates post-absorptively and during insulin infusion at 40 mU m^−2^ min^−1^ based on genotypes for the 3′UTR SNP chr7:44184184-G/A. Black bar, oxidative glucose disposal rate; white bar, non-oxidative glucose disposal rate. Error bar: SD. *p < 0.05; **p < 0.01; ***p < 0.001

Electronic supplementary material (ESM) Fig. 1 shows a flow chart depicting the study design and selection of participants. All individuals in this study are participants of a longitudinal study of the aetiology of type 2 diabetes among the Gila River Indian Community in Arizona, where most of the residents are Pima Indians or Tohono O’odham (a closely related tribe) [8]. Diabetes was determined by prior clinical diagnosis or an oral glucose tolerance test according to the criteria of the American Diabetes Association [9]. A population-based sample of full-heritage Pima Indians (*n* = 3,604, including 736 sibships [sibship is defined as sibs ≥2], Table 1) was initially used to assess associations with type 2 diabetes. A non-overlapping sample of mixed-heritage American Indians from the same longitudinal study (*n* = 4,063, including 739 sibships; reported heritage, on average, was one-half Pima and three-quarters American Indian, Table 1) was used to assess replication. Among these samples, BMI was measured at biennial examinations and maximum BMI observed in the longitudinal study was analysed in 3,391 full-heritage Pima Indians and 3,406 mixed-heritage American Indians (Table 1) who were examined when aged ≥15 years. Fasting serum glucose concentrations were measured in 2,542 full-heritage Pima Indians and 2,887 mixed-heritage American Indians that were non-diabetic, including individuals who subsequently developed diabetes and those who remained non-diabetic (Table 1).

**Table 1:** Characteristics of full-heritage Pima Indians and mixed-heritage American Indians analysed in the population-based association studies

Characteristic	*n*	Male sex (%)	Age (years)	BMI (kg/m^2^)
Full-heritage Pima Indians				
Type 2 diabetes study	3,604			
Diabetic (46%)	1,658	37	49.1 ± 14.1	38.7 ± 8.6
Non-diabetic	1,946	48	32.1 ± 14.6	36.1 ± 8.5
BMI study	3,391	42	36.1 ± 13.3	37.4 ± 8.7
Fasting glucose concentration study	2,542	43	39.0 ± 14.5	35.7 ± 8.3
Mixed-heritage American Indians				
Type 2 diabetes study	4,063			
Diabetic (21%)	853	41	41.1 ± 14.2	38.7 ± 8.7
Non-diabetic	3,210	47	24.9 ± 11.9	33.5 ± 8.4
BMI study	3,406	45	29.2 ± 12.0	34.8 ± 8.8
Fasting glucose concentration study	2,887	44	30.4 ± 12.3	33.5 ± 8.3

Age and BMI data are shown as means ± SD

### Subset of participants with additional inpatient data on quantifiable metabolic traits

Among the full-heritage Pima Indians described above, 415 non-diabetic individuals (including 99 sibships; male sex 58%, age 27 ± 6 years and BMI 34 ± 8 kg/m^2^ at the time of metabolic testing) had undergone detailed studies of metabolic and anthropometric phenotypes for risk factors related to type 2 diabetes and obesity. Body composition, including percentage body fat, fat mass and fat-free mass, was estimated by underwater weighing until 1996 and by dual energy x-ray absorptiometry (DPX-1; Lunar Radiation Corp., Madison, WI, USA ) thereafter [10]. Glucose tolerance was determined by a 75 g OGTT, with measurements of fasting, 30, 60, 120 and 180 min plasma glucose and insulin concentrations [11]. A hyperinsulinaemic–euglycaemic clamp (insulin infusion rate of 40 mU m^−2^ min^−1^ with simultaneous glucose tracers) was used to measure rates of post-absorptive (basal) and insulin-stimulated glucose disappearance as previously described [11]. Indirect calorimetry measurements using a ventilated hood system were performed before and during the insulin infusion to assess rates of energy expenditure and substrate oxidation [12, 13]. Pancreatic beta cell function was assessed by the acute insulin response (AIR) after a 25 g intravenous glucose bolus and calculated as the mean increment in plasma insulin concentrations from 3 to 5 min [11].

To measure 24 h energy expenditure, study participants entered a respiratory chamber for 23 h and 15 min after an overnight fast and after at least 3 days of a weight-maintaining diet [14]. Four meals were provided at 08:00, 11:00, 16:00 and 19:00 hours. Fresh air was drawn through the chamber, and CO_2_ production and O_2_ consumption were measured and calculated every 15 min and extrapolated to the 24 h period [15]. Spontaneous physical activity (SPA) was detected by radar sensors and expressed as percentage of time in motion per 15 min interval. The energy cost of SPA was calculated as the product of average SPA over 24 h and the slope of the regression line between energy expenditure and SPA between 08:00 and 23:00 hours [15]. Sleeping metabolic rate was defined as the average energy expenditure of all 15 min periods between 1:00 and 5:00 hours during which SPA was <1.5%, and was extrapolated to 24 h [16]. The 24 h respiratory quotient (RQ) was calculated as the ratio of 24 h $\overset{\cdot }{V}{\mathrm{CO}}_2$ to 24 h $\overset{\cdot }{V}{\mathrm{O}}_2$. Carbohydrate and lipid oxidation rates were derived from the 24 h RQ after accounting for protein oxidation, which was estimated from the 24 h urinary nitrogen excretion [15].

### Identification and genotyping of single-nucleotide polymorphisms

Single-nucleotide polymorphisms (SNPs) in exons and the putative promoter region (∼1.4 kb upstream of the translational start site) of *GCK* were obtained from whole-genome sequence data (30–40× coverage) of 234 individuals who were predominantly full-heritage Pima Indians (Complete Genomics, Mountain View, CA, USA; Illumina, San Diego, CA, USA). Individuals had been characterised for metabolic traits in our Clinical Research Center and were selected from different nuclear families to maximise identification of genetic variation. Genome sequence data were compared with the reference sequence GRCh37/hg19. SNPs not reported in NCBI dbSNP/1000 genomes (http://www.ncbi.nlm.nih.gov/SNP/; or http://browser.1000genomes.org/) were classified as ‘novel’. Linkage disequilibrium (LD) was determined using Haploview (version 4.2, Broad Institute, Cambridge, MA, USA). Tag SNPs were selected using the Tagger algorithm with a pairwise *r* ^2^ ≥ 0.8 taken as indicative of redundancy.

SNPs were genotyped for association analyses using the TaqMan Allelic Discrimination Assay on an ABI Prism 7900 (Applied Biosystems, Carlsbad, CA, USA) or BeadXpress System (Illumina).

### Allelic specific gene expression

Total RNA was isolated from percutaneous abdominal adipose tissue biopsies from 14 individuals who were heterozygous for the 3′ untranslated region (3′UTR) SNP chr7:44184184-G/A. RNA was reverse-transcribed to cDNA and allelic specific expression was performed on an ABI Prism 7900 using a TaqMan probe (Applied Biosystems). The ratio of allele G/A expression was normalised to the genomic DNA control.

### Statistical analyses

Statistical analyses were performed using the software of the SAS Institute (version 9.2, Cary, NC, USA). A logistic regression analysis was used to assess the association of genotypes with type 2 diabetes and was adjusted for age, sex, birth year and heritage as covariates. The model was fit with the generalised estimating equation (GEE) to account for dependence among siblings. Genotype was analysed as a numeric variable representing 0, 1 or 2 copies of a given allele. To estimate the proportion of European ancestry, 45 informative markers with large differences in allele frequency between populations [17] were used as a covariate in these analyses. The association of quantifiable traits with genotypes was analysed by linear regression using the GEE procedure to account for correlation among siblings. Results were adjusted for covariates as indicated. The rate of carbohydrate oxidation, as a metabolic predictor of diabetes, was assessed in individuals with normal glucose tolerance (NGT) who had measures of carbohydrate oxidation rate at baseline during a hyperinsulinaemic–euglycaemic clamp. These individuals also had biennial follow-up OGTTs to determine diabetes status. The Cox proportional hazards model was used to determine the hazard rate ratio (HRR) for developing type 2 diabetes associated with the rate of insulin-stimulated carbohydrate oxidation including age, sex, percentage body fat, AIR and non-oxidative glucose disposal rate as covariates. Follow-up time was defined as the time from the measure of carbohydrate oxidation rate during an insulin clamp to either type 2 diabetes onset or the last evaluation when an individual remained non-diabetic. To analyse the effect of genotype on the energy expenditure trajectory, a mixed model analysis was used including time, time^2^ and time^3^ as fixed effects to model the non-linearity of the trajectory. Results were adjusted for age, sex, fat mass, fat-free mass and SPA.

## Results

### Association of GCK SNPs with carbohydrate oxidation rate

Whole-genome sequence data from 234 Pima Indians were used to identify common SNPs (mAF ≥ 0.01). Five SNPs were identified in exon regions: two synonymous amino acid substitutions, GCK-G193G and GCK-Y215Y (rs144723656), and three 3′-UTR SNPs, rs13306388, rs55714218 and a novel SNP chr7:44184184-G/A. Four SNPs were identified in the putative promoter region and were predicted to cause loss of function by the Ingenuity Variant Analysis (https://variants.ingenuity.com): rs1799831, rs1799884, rs193226243 and rs1476891. These nine SNPs and rs4607517 (which has been associated with type 2 diabetes in other studies [5–7] and maps 6.6 kb upstream of *GCK*) were analysed for pairwise LD. Eight tag SNPs capture all ten SNPs (*r* ^2^ ≥ 0.8, ESM Fig. 2).

These eight tag SNPs (mAF ≥ 0.01) were genotyped in a population-based sample of 3,604 full-heritage Pima Indians of which 415 non-diabetic individuals had detailed measures of metabolic and anthropometric phenotypes for risk factors related to type 2 diabetes and obesity. Associations of these eight tag SNPs with metabolic traits were analysed in these 415 individuals. For the novel 3′UTR SNP chr7:44184184-G/A (mAF 0.05), only two of the 415 individuals were homozygous for the minor A allele, so their data were combined with those of the G/A heterozygotes for statistical analyses. Individuals with the A allele had a lower mean rate of basal carbohydrate oxidation (Fig. 1, *β* = 0.22 mg [kg estimated metabolic body size (EMBS)]^−1^ min^−1^ per risk allele, *p* = 0.005, adjusted for age, sex and percentage body fat) and lower rate of insulin-stimulated carbohydrate oxidation (*β* = 0.24 mg [kg EMBS]^−1^ min^−1^, adjusted *p* = 0.0002) when compared with individuals with the G allele. However, the non-oxidative glucose disposal rate at baseline and during insulin stimulation was not different between the two groups (adjusted *p* = 0.18 at basal state, *p* = 0.92 during insulin stimulation).

Furthermore, individuals with the A allele had a higher lipid oxidation rate at baseline by 0.08 mg [kg EMBS]^−1^ min^−1^ (Table 2, *p* = 0.007, adjusted for age, sex and percentage body fat) and during insulin infusion by 0.09 mg [kg EMBS]^−1^ min^−1^ (adjusted *p* = 0.01) when compared with individuals with the G allele. These changes in lipid oxidation rate may be secondary to the changes in carbohydrate oxidation rate. Individuals with the A allele also had a lower basal rate of endogenous glucose production (*β* = 0.08 mg [kg EMBS]^−1^ min^−1^, adjusted *p* = 0.03), but no difference in endogenous glucose production rate during insulin infusion (adjusted *p* = 0.31). This 3′UTR SNP was not associated with the whole-body insulin-stimulated glucose disposal rate (adjusted *p* = 0.19). The resting metabolic rate also did not differ (*p* = 0.94, adjusted for age, sex, fat mass and fat-free mass) between those with the A allele and those with the G allele.

**Table 2:** Metabolic characteristics of non-diabetic full-heritage individuals by genotypes of the 3′UTR SNP chr7:44184184-G/A

Characteristic	chr7:44184184 (mean ± SD)			*p* value^a^
	G/G	G/A + AA	Estimate	
Participants (*n*)	369	33		
Body fat (%)	32.9 ± 8.6	34.0 ± 7.9	−0.82	0.54
Oral glucose tolerance test				
Fasting plasma glucose (mmol/l)	4.4 ± 0.5	5.0 ± 0.8	−0.05	0.68
2 h plasma glucose (mmol/l)	6.9 ± 1.7	6.9 ± 1.8	−0.15	0.56
Log_10_ fasting plasma insulin (pmol/l)	3.0 ± 1.1	3.0 ± 1.1	0.87	0.67
Log_10_ 2 h plasma insulin (pmol/l)	2.4 ± 1.0	2.4 ± 1.0	0.86	0.93
Hyperinsulinaemic– euglycaemic clamp (mg [kg EMBS]^−1^ min^−1^)^b^				
Log_10_ glucose disposal rate	0.55 ± 0.1	0.51 ± 0.1	0.02	0.19
Carbohydrate oxidation	2.10 ± 0.5	1.76 ± 0.5	0.24	0.0002
Lipid oxidation	0.41 ± 0.3	0.55 ± 0.3	−0.09	0.01
Endogenous glucose output	0.38 ± 0.4	0.33 ± 0.3	0.05	0.31
Basal glucose output (mg [kg EMBS]^−1^ min^−1^)	1.91 ± 0.2	1.83 ± 0.2	0.08	0.03
Basal carbohydrate oxidation (mg [kg EMBS]^−1^ min^−1^)	1.42 ± 0.4	1.20 ± 0.5	0.22	0.005
Basal lipid oxidation (mg [kg EMBS]^−1^ min^−1^)	0.71 ± 0.3	0.80 ± 0.2	−0.08	0.007
Resting metabolic rate (kJ/day)	7,314 ± 1,298	7,574 ± 1,583	−8.08	0.94
Participants, NGT (*n*)	268	23		
Log_10_ AIR (pmol/l)	3.2 ± 1.1	3.2 ± 1.2	0.91	0.46
Log_10_ 30-min plasma insulin (pmol/l)	3.2 ± 1.1	3.2 ± 1.1	0.89	0.43
Participants in metabolic chamber study (*n*)	277	23		
Body fat (%)	33.2 ± 8.2	33.9 ± 7.6	0.08	0.99
24 h RQ	0.85 ± 0.02	0.85 ± 0.02	0.004	0.53
Carbohydrate oxidation (kJ/day)	4,589 ± 988	4,363 ± 959	311	0.03
Lipid oxidation (kJ/day)	4,007 ± 1,269	3,866 ± 1,034	134	0.61
Protein oxidation (kJ/day)	1,231 ± 536	1,281 ± 477	−60	0.53
24 h energy expenditure (kJ/day)	9,927 ± 1,700	9,734 ± 1,486	520	3.39 × 10^−6^
Sleeping metabolic rate (kJ/day)	7,046 ± 1,214	7,138 ± 1,017	136	0.27
Energy cost of SPA (kJ/day)	1,562 ± 565	1,206 ± 494	164	0.16

Values for mean ± SD were unadjusted. The effect size estimates were calculated as the differences in least square means and were adjusted for covariates

Rate of glucose disappearance during insulin stimulation, fasting, 30 min and 2 h plasma insulin concentrations and AIR were log_10_-transformed before analyses to approximate a normal distribution

^a^The *p* value for percentage body fat was adjusted for age and sex. The *p* value for AIR was adjusted for age, sex, percentage body fat and rate of glucose disappearance during insulin stimulation. The *p* values for resting metabolic rate and sleeping metabolic rate were adjusted for age, sex, fat mass and fat-free mass. The *p* value for 24 h energy expenditure was adjusted for age, sex, fat mass, fat-free mass and SPA. The *p* values for 24 h RQ and macronutrient oxidation were adjusted for age, sex, percentage body fat and energy balance. All remaining *p* values were adjusted for age, sex and percentage body fat

^b^EMBS is equivalent to fat-free mass + 17.7 kg

### Association of GCK SNPs with 24 h energy expenditure

**Fig. 2:**
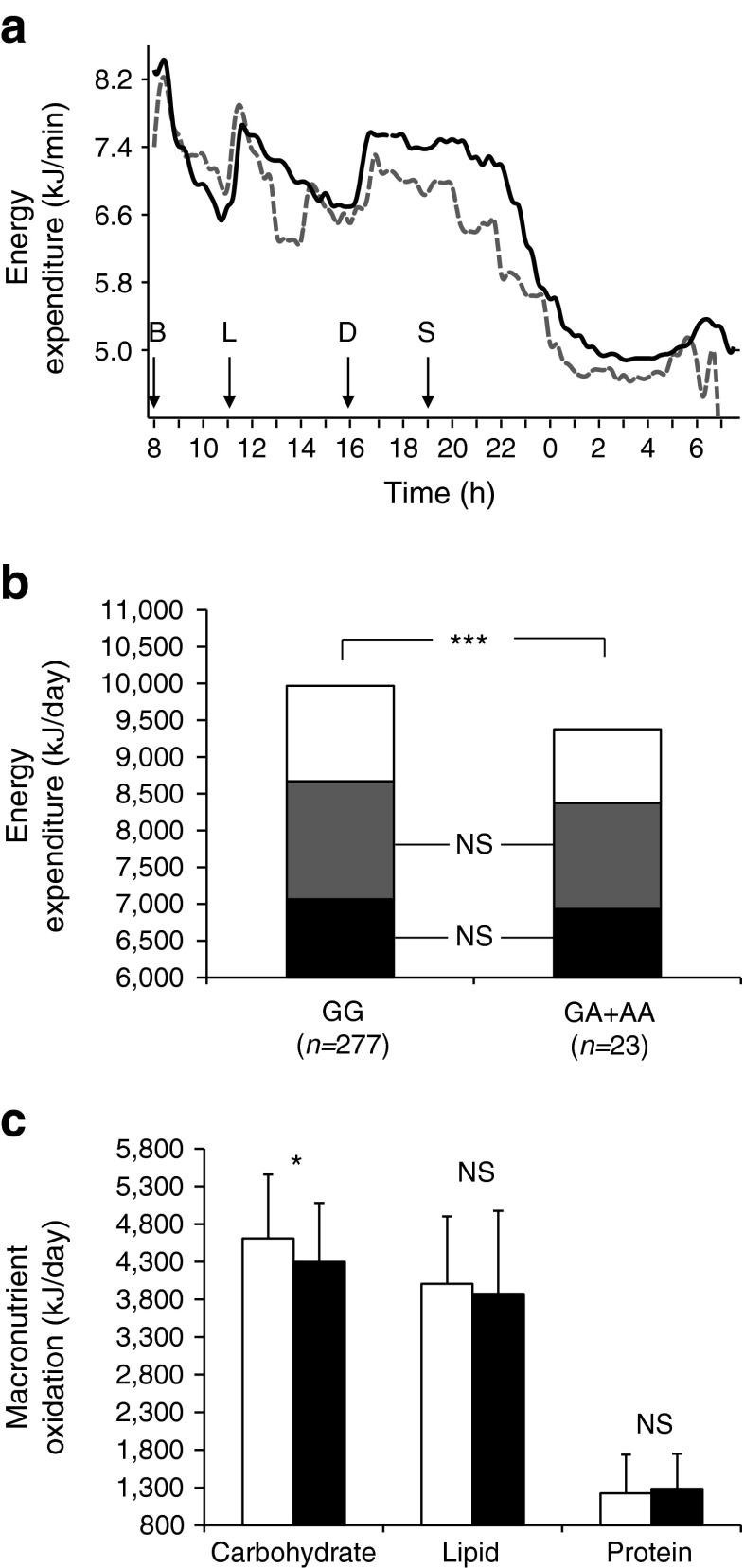
(**a**) Time course of 24 h energy expenditure in the respiratory chamber based on genotypes for the 3′UTR SNP chr7:44184184-G/A. Arrows indicate when meals were provided (B, breakfast; L, lunch; D, dinner; S, snack); solid line, homozygous GG (*n* = 251); dashed line, GA + AA (*n* = 22). (**b**) Components of 24 h energy expenditure based on genotypes for the 3′UTR SNP. White bar, the thermic effect of food; grey bar, SPA; black bar, sleeping metabolic rate. ***p < 0.001. (**c**) 24 h macronutrient oxidation in the respiratory chamber based on genotype for the 3′UTR SNP. White bar, GG (*n* = 277); black bar, GA + AA (*n* = 23). Error bar: SD. *p < 0.05

In the respiratory chamber study, participants with the A allele for the 3′UTR SNP chr7:44184184-G/A had a lower 24 h energy expenditure (by 520 kJ/day) than those with the G allele (as the difference in least square means); this was more evident in the postprandial state, as shown by the energy expenditure trajectory over the day (Fig. 2a).

Three components of 24 h energy expenditure, including the sleeping metabolic rate (60–70% of total energy expenditure), energy cost of SPA (20–30%) and thermic effect of food (awake and fed thermogenesis, 10%) were described previously [15, 18]. The difference in 24 h energy expenditure between genotypes of the 3′UTR SNP is attributable to the difference in the thermic effect of food (Fig. 2b, *β* = 520 kJ/day, *p* = 3.39 × 10^−6^ for 24 h energy expenditure, adjusted for age, sex, fat mass, fat-free mass and SPA), but not to SPA (*p* = 0.16, adjusted for age and sex) or sleeping metabolic rate (adjusted for age, sex, fat mass and fat-free mass, *p* = 0.27). To confirm this observation, we further analysed the effects of genotype on the energy expenditure trajectory during the postprandial state (daytime) vs fasting state (night-time) using a mixed model analysis. After accounting for age, sex, fat mass, fat-free mass and SPA, individuals with the A allele had a lower rate of energy expenditure during the day (*β* = −0.46 kJ/min, *p* = 0.0001, from 8:00 hours on one day to 01:00 hours on the next day), compared with those with the G allele, but no difference was observed during the night (*β* = −0.07 kJ/min, *p* = 0.50, from 01:00 hours to 05:00 hours). This result demonstrates that the difference in 24 h energy expenditure between genotypes is driven by the thermic effect of food in the postprandial state.

In the chamber, individuals with the A allele also had a decreased rate of carbohydrate oxidation (by 311 kJ/day) compared with those with the G allele (Fig. 2c, *p* = 0.03, adjusted for age, sex, percentage body fat and energy balance). This was comparable with the difference in the rate of carbohydrate oxidation during a hyperinsulinaemic–euglycaemic clamp, in which a ∼70 kg (EMBS) individual with the A allele was estimated to have a lower rate of insulin-stimulated carbohydrate oxidation (by ∼406 kJ/day). However, neither the rate of lipid oxidation nor protein oxidation differed between genotypes (*p* = 0.61 and *p* = 0.53, respectively, adjusted for age, sex, percentage body fat and energy balance), indicating that the 3′UTR SNP affected energy expenditure primarily via carbohydrate oxidation.

All eight tag SNPs were analysed for associations with metabolic traits in 415 full-heritage non-diabetic individuals (data not shown). Only the 3′UTR SNP was associated with the rate of carbohydrate oxidation and 24 h energy expenditure (Figs 1 and 2, Table 2).

### Association of GCK SNPs with type 2 diabetes and BMI

The eight tag SNPs were genotyped in a population-based sample of 3,604 full-heritage Pima Indians and a replication sample of 4,063 mixed-heritage American Indians for association analyses of type 2 diabetes and BMI. The 3′UTR SNP chr7:44184184-G/A had a nominal association with type 2 diabetes in full-heritage Pima Indians (Table 3; OR 1.37, 95% CI 1.06, 1.78, *p* = 0.015, adjusted for age, sex, birth year and heritage). This SNP also had a borderline association with type 2 diabetes in a replication sample of mixed-heritage American Indians (OR 1.32, 95% CI 0.97, 1.79, adjusted *p* = 0.075). A lower *p* value was observed in a combined analysis of all individuals (OR 1.36, 95% CI 1.11, 1.65, adjusted *p* = 0.002, *n* = 7,667). Due to the low allele frequency (mAF 0.05), hence limited statistical power, this SNP only achieved a type 2 diabetes association at *p* = 0.002 despite a considerable effect size (OR 1.36). The A allele, associated with a lower rate of carbohydrate oxidation rate and 24 h energy expenditure, was associated with a higher prevalence of type 2 diabetes. In addition, the promoter SNP rs1476891 (*D*′ = 0.88, *r* ^2^ = 0 with the 3′UTR SNP, ESM Fig. 2) had a nominal association with type 2 diabetes in a combined analysis (OR 1.15, 95% CI 1.02, 1.31, adjusted *p* = 0.03).

**Table 3:** Associations of eight tag SNPs in *GCK* with type 2 diabetes and fasting glucose concentrations in American Indians

SNP	Risk/Non	Full-heritage, type 2 diabetes (*n* = 3,604)			Mixed-heritage, type 2 diabetes (*n* = 4,063)			Combined, type 2 diabetes (*n* = 7,667)		Combined, fasting glucose (*n* = 5,429)	
		RAF	OR (95% CI)	*p* value	RAF	OR (95% CI)	*p* value	OR (95% CI)	*p* value	β value	*p* value
rs4607517^a^ 5′-upstream	T/C	0.34	0.97 (0.86, 1.09)	0.616	0.29	1.20 (1.04, 1.38)	0.010	1.07 (0.97, 1.17)	0.158	0.06	8.7 × 10^−7^
rs1476891 promoter	A/G	0.10	1.26 (1.04, 1.53)	0.016	0.16	1.08 (0.91, 1.29)	0.380	1.15 (1.02,1.31)	0.029	0.03	0.053
rs193226243 promoter	A/G	0.09	1.11 (0.91, 1.36)	0.316	0.06	0.97 (0.72, 1.30)	0.837	1.07 (0.90, 1.26)	0.445	0.08	0.0003
rs1799831 promoter	T/C	0.94	1.02 (0.79, 1.31)	0.894	0.89	0.82 (0.68, 1.00)	0.056	0.91 (0.77, 1.06)	0.215	0.01	0.476
GCK-G193G Gly193Gly	C/T	0.01	1.17 (0.68, 2.01)	0.581	0.01	1.98 (0.97, 4.08)	0.061	1.38 (0.86, 2.20)	0.177	0.25	4.3 × 10^−5^
rs13306388 3′UTR	C/T	0.74	1.04 (0.91, 1.19)	0.603	0.76	1.04 (0.89, 1.22)	0.605	1.04 (0.94, 1.15)	0.470	0.01	0.516
chr7:44184184 3′UTR	A/G	0.05	1.37 (1.06, 1.78)	0.015	0.03	1.32 (0.97, 1.79)	0.075	1.36 (1.11, 1.65)	0.002	−0.02	0.473
rs55714218 3′UTR	G/-	0.78	1.05 (0.92, 1.20)	0.462	0.71	0.84 (0.73, 0.97)	0.014	0.95 (0.86, 1.05)	0.292	−0.01	0.515

The analysis for ‘combined’ is conducted in full-heritage Pima Indians and mixed-heritage American Indians. The risk allele (given first) for rs4607517 is defined as the observed risk allele in European studies, while for other SNPs it is defined as the allele with a higher risk of diabetes in full-heritage Pima Indians; ORs are given per copy of this allele. RAF is the frequency of the risk allele. Beta for fasting glucose concentrations represents the effect in mmol/l per copy of the risk allele. The *p* values were adjusted for age, sex, birth year and heritage

^a^Established type 2 diabetes variant in European populations

SNP rs4607517, which was reproducibly associated with fasting glucose concentrations in other populations [5–7], also had strong associations with fasting glucose concentrations in a combined analysis of 5,429 American Indians (*β* = 0.06 mmol/l per risk allele, *p* = 8.7 × 10^−7^, adjusted for age, sex, birth year and heritage) (Table 3). However, this SNP was not associated with type 2 diabetes (adjusted *p* = 0.16, *n* = 7,667). In addition, the synonymous SNP Gly193Gly and rs193226243 were also associated with fasting glucose concentrations (*β* = 0.25 mmol/l, adjusted *p* = 4.3 × 10^−5^; *β* = 0.08 mmol/l, adjusted *p* = 0.0003, respectively).

Beta cell function was assessed by AIR in 298 full-heritage Pima Indians with NGT. SNP rs4607517, associated with fasting glucose concentrations, was nominally associated with AIR (*β* = 0.89 [log_10_ scale], *p* = 0.07, adjusted for age, sex, percentage body fat and rate of glucose disappearance during insulin stimulation). The promoter SNP rs193226243 was also associated with AIR (*β* = 0.92 [log_10_ scale], adjusted *p* = 0.02). However, the 3′UTR SNP was not associated with AIR (adjusted *p* = 0.46).

Association of the eight tag SNPs with maximum BMI were also analysed (Table 4). SNP rs193226243 had a nominal association with BMI in a combined analyses of 6,797 American Indians (*β* = 0.02 (log_e_ scale), *p* = 0.008, adjusted for age, sex, birth year and heritage). None of other SNPs was consistently associated with BMI.

**Table 4:** Associations of eight tag SNPs in *GCK* with maximal BMI in full-heritage Pima Indians, mixed-heritage American Indians and the combined samples

SNP	Risk/Non	Full-heritage Pima Indian (*n* = 3,391), mean BMI (kg/m^2^)						Mixed-heritage American Indian (*n* = 3,406), mean BMI (kg/m^2^)						Combined (*n* = 6,797)	
		RAF	Risk/Risk	Risk/Non	Non/Non	*β* value	*p* value	RAF	Risk/Risk	Risk/Non	Non/Non	*β* value	*p* value	*β* value	*p* value
rs4607517	T/C	0.34	38.2	37.5	37.2	0.013	0.028	0.29	34.8	35.1	34.7	0.003	0.608	0.008	0.065
rs1476891	A/G	0.10	37.7	36.9	37.6	−0.006	0.512	0.16	34.3	34.4	35.0	−0.002	0.781	−0.005	0.433
rs193226243	A/G	0.09	39.2	37.9	37.4	0.012	0.216	0.06	30.7	36.4	34.6	0.029	0.020	0.020	0.008
rs1799831	T/C	0.94	37.5	36.9	38.1	0.012	0.307	0.89	34.9	34.8	30.4	0.005	0.555	0.009	0.238
GCK-G193G	C/T	0.01		38.7	37.4	0.046	0.085	0.01		35.1	34.8	−0.028	0.548	0.020	0.432
rs13306388	C/T	0.74	37.3	37.7	38.2	−0.006	0.342	0.76	34.8	34.7	34.6	−0.002	0.720	−0.005	0.321
Chr7:44184184	A/G	0.05	41.4	37.9	37.4	0.008	0.571	0.03	30.8	36.6	34.7	0.018	0.268	0.012	0.254
rs55714218	G/−	0.78	37.8	37.7	37.1	0.002	0.822	0.71	34.7	34.8	34.7	0.002	0.728	0.003	0.555

BMI is the maximum value observed in the longitudinal study from all examinations after age 15 years. The risk allele is defined as the allele with a higher risk of diabetes in full-heritage Pima Indians; the regression coefficient (B) represents the effect on the logarithmic scale (log_e_) per copy of the risk allele. Mean BMI was unadjusted. The *p* values were adjusted for age, sex, birth year and heritage

### The predictive effect of carbohydrate oxidation rate on development of type 2 diabetes

**Fig. 3:**
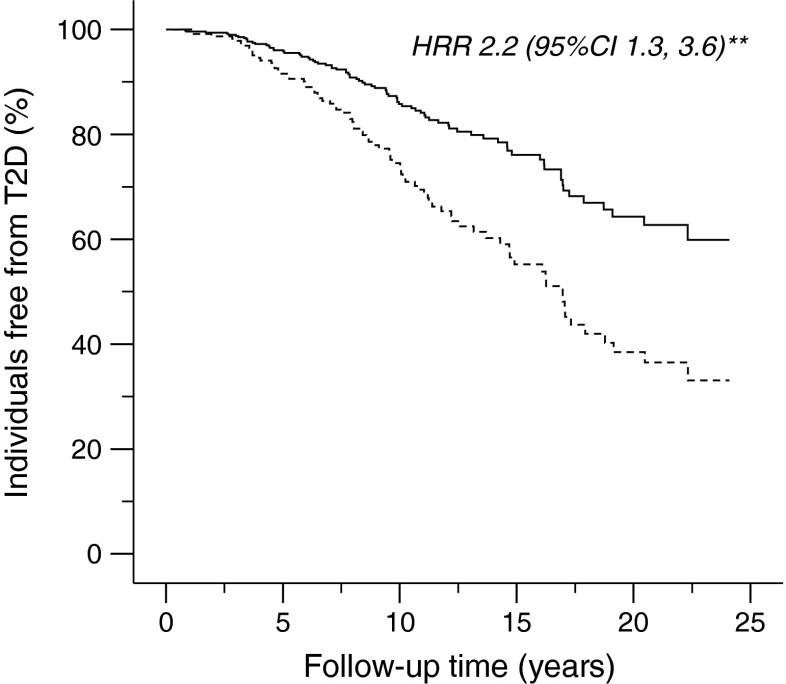
Survival curve for time to type 2 diabetes (T2D) onset in 287 full-heritage Pima Indians with NGT at baseline. Solid line, carbohydrate oxidation rate > a mean of 1.63 mg (kg EMBS)^−1^ min^−1^ (*n* = 137); dashed line, carbohydrate oxidation rate <1.63 mg (kg EMBS)^−1^ min^−1^ (*n* = 150). Data are plotted up to the follow-up time of ∼23 years, and omitted at longer follow-up time when only 1% of participants were involved. HRR 2.2, 95% CI 1.3, 3.6; **p < 0.01

We further evaluated the relationship between the rate of insulin-stimulated carbohydrate oxidation and the risk of developing type 2 diabetes in 287 full-heritage Pima Indians. Of these 287 individuals with NGT who had measures of carbohydrate oxidation rate during insulin stimulation at baseline (baseline age 26.4 ± 6.0 years) and also had follow-up data for development of diabetes (follow-up time 7.8 ± 8.2 years), 99 (34%) developed type 2 diabetes. Figure 3 shows the Kaplan–Meier survival curve for time to type 2 diabetes onset with participants categorised as those with a higher or lower rate of insulin-stimulated carbohydrate oxidation than the mean of 1.63 mg (kg EMBS)^−1^ min^−1^. Individuals with a lower rate of insulin-stimulated carbohydrate oxidation (*n* = 150; carbohydrate oxidation rate 1.35 ± 0.23 mg [kg EMBS]^−1^ min^−1^; age 26.4 ± 5.8 years) had a shorter period to type 2 diabetes onset, hence a higher risk for developing type 2 diabetes as compared with those with a higher rate of carbohydrate oxidation (*n* = 137; carbohydrate oxidation rate 1.94 ± 0.26 mg [kg EMBS]^−1^ min^−1^; age 26.5 ± 6.3 years) (HRR 2.2, 95% CI 1.3, 3.6, *p* = 0.002, adjusted for age, sex, percentage body fat, AIR and non-oxidative glucose disposal rate). The same result was observed with individuals categorised as those with a higher or lower median rate of insulin-stimulated carbohydrate oxidation (1.61 mg [kg EMBS]^−1^ min^−1^). In a Cox proportional hazards analysis, a lower than the mean rate of insulin-stimulated non-oxidative glucose disposal at baseline also predicted a higher risk for developing type 2 diabetes than a rate above the mean (HRR 2.5, 95% CI 1.4, 4.5, *p* = 0.002, adjusted for age, sex, percentage body fat, AIR and glucose oxidation rate; data not shown).

### Allelic specific GCK expression

To investigate whether the alleles of the 3′UTR SNP chr7:44184184-G/A differentially influence gene expression, allelic specific expression of *GCK* was assessed in adipose tissue biopsies from individuals heterozygous for this SNP. No difference in the allelic expression of *GCK* was observed in this tissue (G/A = 0.993 vs expected ratio of 1, *p* = 0.2, data not shown). Since liver and pancreas tissue biopsies from Pima Indians were not available for study, the allelic specific expression of *GCK* in these tissues is not known.

## Discussion

GCK is the main glucose-phosphorylating enzyme in the liver and pancreatic beta cells. It converts glucose to G6P as a first and rate-limiting step in glycolysis, which plays a part in the process of glucose oxidation [1–3]. Our study indicates that a novel variant in the 3′UTR of *GCK*, with a risk allele frequency of 0.05, is associated with a lower rate of glucose oxidation post-absorptively, during insulin stimulation and after a diet of mixed consumption, which is in agreement with the role of GCK in glycolysis. This variant was not associated with non-oxidative glucose disposal, suggesting that glucose storage (glycogen synthesis) was not affected. It is known that rare mutations in *GCK* occurring in MODY result from a reduced glucose-stimulated insulin secretion. Although rs193226243 and rs4607517 had borderline associations with AIR, none of the other common variants were associated with AIR. Therefore, our data suggest that common variation in *GCK* predominantly influences glycolysis and the rate of glucose oxidation in hepatocytes. These data are consistent with the observations that overexpression of *GCK* in mouse liver or rat isolated hepatocytes enhances glucose oxidation [19, 20]. Nevertheless, a subtle effect of common *GCK* variants on beta cell function cannot be ruled out. Since the *GCK* variants affect the threshold for glucose sensing, the effect size and/or sample size may be too small to render a statistical difference in insulin secretion. It is also possible that the 25 g intravenous glucose bolus used in the AIR measurement may be above the threshold at which GCK exerts its effect, thus limiting a positive detection.

Hepatic GCK serves as a major component of the hepatic glucose-sensing system involved in glucose synthesis, breakdown and storage. While glycolysis and glycogen synthesis pathways are activated during the postprandial state, gluconeogenesis and glycogen breakdown are involved in hepatic glucose production in the post-absorptive state. Our data indicate that the association of the 3′UTR *GCK* variant with the rate of basal hepatic glucose production is likely due to an association with the rate of basal glucose oxidation. However, this variant was not associated with hepatic glucose production during insulin stimulation, despite previous findings demonstrating that a *GCK* variant was associated with hepatic insulin resistance [21].

In addition to its pivotal role in glucose metabolism, a new role for hepatic GCK in energy metabolism has emerged in recent studies. Tsukita and co-workers reported that upregulation of hepatic *GCK* by high-fat diet feeding in mice suppresses brown adipose tissue (BAT) thermogenesis via leptin-mediated neural signals and downregulation of uncoupling protein-1 [22, 23]. This GCK-mediated liver-to-BAT neuronal relay system provides a novel mechanism in modulating obesity predisposition in mice. In this study, we report that the novel 3′UTR variant in *GCK* had a significant effect on 24 h energy expenditure through a change in the thermic effect of food. This effect resulted from a change in the rate of carbohydrate oxidation rather than from any apparent effect on BAT thermogenesis.

Measures of energy expenditure, thermic effect of food and substrate oxidation are predictors of weight change. In Pima Indians, individuals with lower than expected energy expenditure are at higher risk for future long-term increases in weight and fat mass [16]. The thermic effect of food is also reduced in obese individuals and predicts their future weight gain [18]. A higher rate of insulin-stimulated carbohydrate oxidation during a hyperinsulinaemic–euglycaemic clamp predicts a future weight again [24]. The rate of carbohydrate oxidation in a respiratory chamber also predicts short-term changes in body weight [14], but not long-term changes [16]. In the present study, the 3′UTR SNP, associated with rates of energy expenditure and carbohydrate oxidation, was not associated with BMI. Nevertheless, this 3′UTR SNP was associated with risk of type 2 diabetes in Pima Indians. This most likely results from the effect on carbohydrate oxidation since we found that a lower carbohydrate oxidation rate during insulin stimulation was associated with a higher risk of type 2 diabetes, independent of age, sex, percentage body fat, AIR and non-oxidative glucose disposal rate. Thus, while rare mutations in *GCK* cause MODY and neonatal diabetes, our data indicate that common variation in *GCK* with a modest effect on the rate of carbohydrate oxidation contributes to risk of type 2 diabetes.

In summary, our study in individuals who had been extensively characterised for metabolic traits provides cohesive evidence to support a hepatic effect of a novel 3′UTR variant in *GCK* on influencing carbohydrate oxidation, energy expenditure and type 2 diabetes risk; this is consistent with the role of GCK in hepatic glycolysis and energy metabolism. However, our functional analysis of this 3′UTR SNP in adipose tissue did not support a role in allelic imbalance of *GCK* expression in this particular tissue. Interpretation of this negative result is unclear since this SNP could potentially affect transcriptional regulation or mRNA stability in a tissue-specific manner, and we do not have access to liver or pancreatic beta cells from Pima individuals. Alternatively, this 3′UTR SNP might alter GCK translation via an effect on microRNA binding, or perhaps this SNP is in LD with an undiscovered functional variant. Future studies in liver or pancreatic biopsy tissues would clarify some of these possible mechanisms.

## Electronic supplementary material

Below is the link to the electronic supplementary material.

ESM Fig. 1 (PDF 249 kb)

ESM Fig. 2 (PDF 85 kb)

## Abbreviations

3′UTR: 3′ Untranslated region
AIR: Acute insulin response
BAT: Brown adipose tissue
EMBS: Estimated metabolic body size
G6P: Glucose-6-phosphate
GCK: Glucokinase
GEE: Generalised estimating equation
HRR: Hazard rate ratio
mAF: Minor allele frequency
LD: Linkage disequilibrium
NGT: Normal glucose tolerance
RQ: Respiratory quotient
SNP: Single-nucleotide polymorphism
SPA: Spontaneous physical activity
