# Supplementary figures and images for: Common genetic variation in the glucokinase gene (GCK) is associated with type 2 diabetes and rates of carbohydrate oxidation and energy expenditure
Source: Diabetologia. 2014 Apr 13;57(7):1382–90. doi: 10.1007/s00125-014-3234-8 (PMC4052004; doi:10.1007/s00125-014-3234-8)

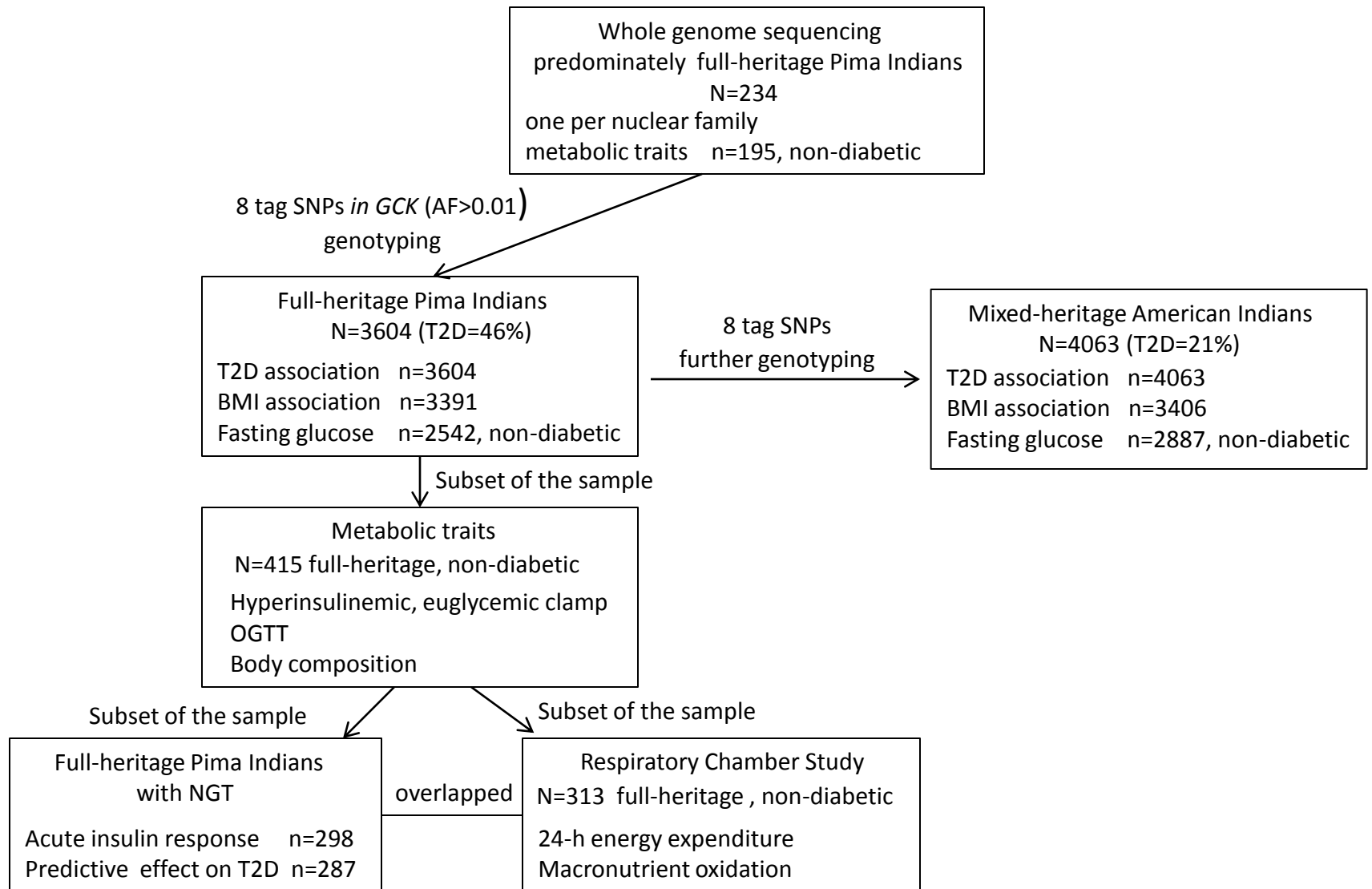

ESM Fig. 1 Flow chart depicting the study design and selection of participants

Supplement: Supplementary file 1 — (PDF 249 kb) [file 125_2014_3234_MOESM1_ESM.pdf]
