# Supplementary material for: Common genetic variation in the glucokinase gene (GCK) is associated with type 2 diabetes and rates of carbohydrate oxidation and energy expenditure
Source: Diabetologia. 2014 Apr 13;57(7):1382–90. doi: 10.1007/s00125-014-3234-8 (PMC4052004; doi:10.1007/s00125-014-3234-8)
Supplement: Supplementary file 2 — (PDF 85 kb) [file 125_2014_3234_MOESM2_ESM.pdf]

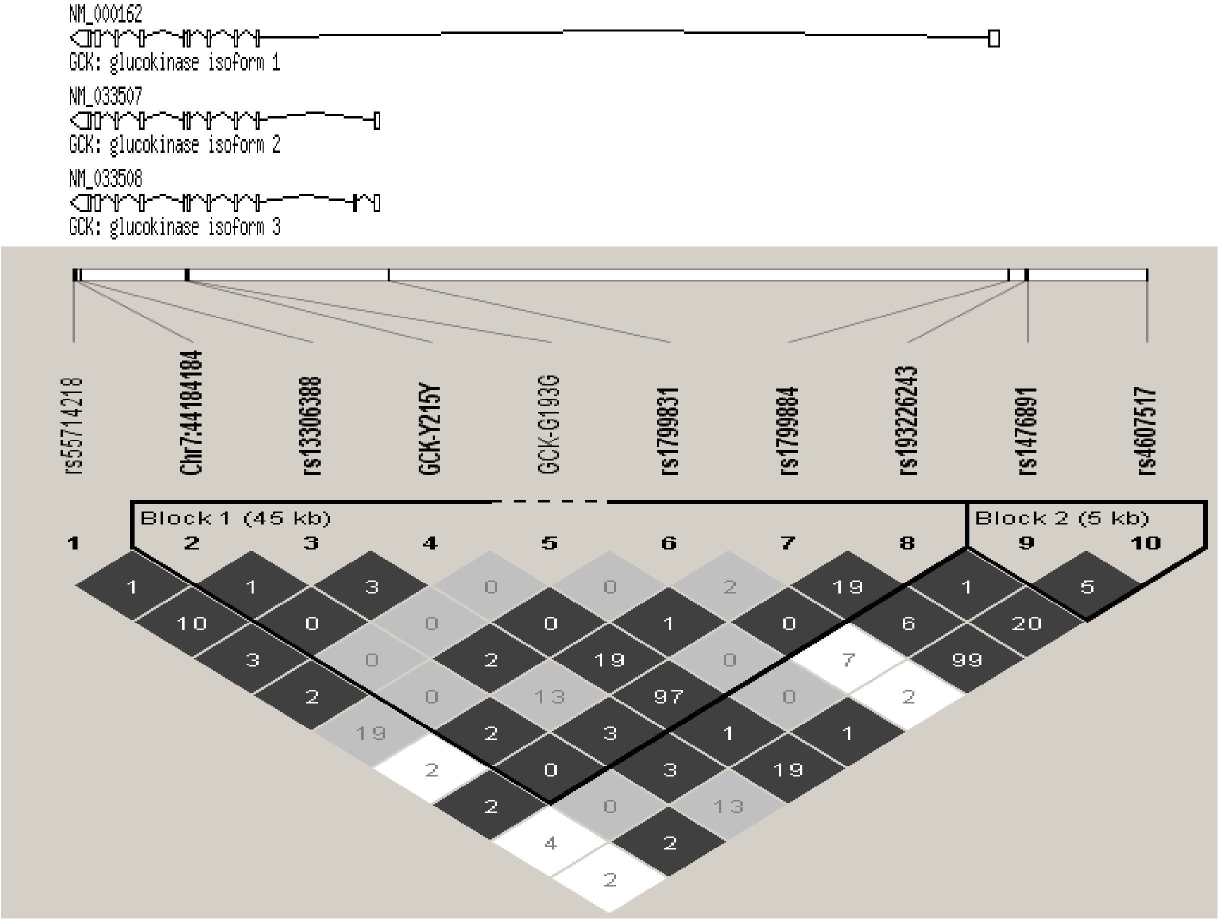

ESM Fig. 2 Relative positions and pair-wise linkage disequilibrium of ten *GCK* SNPs in full-heritage Pima Indians. Haplotype block was determined using default confidence interval algorithm implemented in Haploview 4.2. A block was created if 95% of informative comparisons were "strong LD", ignoring markers with minor allele frequency<0.05. LD (D') is displayed as the confidence bounds of color scheme where dark gray represents "strong evidence of LD", light gray represents "uninformative" and white represents "strong recombinant". The values in the box r2 indicate redundancy. Eight tag SNPs were determined ($r2 \geq 0.8$) as following: rs4607517 (tagging rs1799884), rs1476891, rs193226243 (tagging GCK-Y215Y), rs1799831, GCK-G193G, rs13306388, chr7:44184184 and rs55714218
